# Botnet dataset with simultaneous attack activity

**DOI:** 10.1016/j.dib.2022.108628

**Published:** 2022-09-24

**Authors:** Muhammad Aidiel Rachman Putra, Dandy Pramana Hostiadi, Tohari Ahmad

**Affiliations:** a Department of Informatics*,* Institut Teknologi Sepuluh Nopember*,* Surabaya*,* Indonesia; b Department of Informatics*,* Institut Teknologi Dan Bisnis STIKOM Bali*,* Bali, Indonesia

**Keywords:** Botnet dataset, Bot group activities, Bot communication behavior, Infrastructure, Network security

## Abstract

The proposed dataset shows characteristics of simultaneous botnet attack activities. Botnet network traffic has sequentially interconnected as formed as bidirectional network flow (binetflow), which is combined with normal activities. The dataset is generated from a simulation process by extracting botnet pattern behaviors taken from CTU-13 and NCC datasets. The extraction results are utilized as the basis for simulations to produce a new dataset with simultaneous botnet attack activities. The term “simultaneous attack activities” refers to an attack activity that involves multiple botnets and happens at the same time. The dataset contains several botnet types distributed over three detection sensors. Each dataset has 18 network header features with a total recording duration of 8 h. The bot attack spreads must be appropriately handled by efficient processing, also known as parallel computation detection.

**Specifications Table**

**Table:** 

Subject	Cryptography and Cybersecurity
Specific subject area	Anomaly detection, Botnet, Cyber security*,* Intrusion Detection System
Type of data	Binetflow
How the data were acquired	The dataset is obtained by simulating botnet attacks on the network and extracting activity patterns of CTU-13 and NCC datasets. The attack pattern refers to any sequentially related attack activity combined with normal traffic. The simulation process is developed using Python programming language.
Data format	Raw
Description of data collection	The dataset is a botnet attack simulation based on data extracted from the CTU-13 and NCC datasets, producing a botnet dataset with simultaneous attack characteristics. Botnet communication patterns are classified as distributed, centralized, and spread activities. The dataset consists of various botnet types distributed in three detection sensors. Each dataset has 18 features as a network header.
Data source location	*Institution*: Institut Teknologi Sepuluh Nopember *City/Town/Region*: Surabaya *Country*: Indonesia
Data accessibility	Repository name: Mendeley Direct URL to data: https://doi.org/10.17632/8dpt85jrhp.2 Repository name: Zenodo Direct URL to source code: https://doi.org/10.5281/zenodo.6852833
Related research article	M.A.R. Putra, T. Ahmad, D.P. Hostiadi, Analysis of Botnet Attack Communication Pattern Behavior on Computer Networks, Int. J. Intell. Eng. Syst. 15 (2022). https://doi.org/10.22266/ijies2022.0831.48

## Value of the Data

• The dataset represents botnet attack activities, consisting of those associated sequentially with simultaneous activities detected on multiple parallel sensors. This has overcome the lack of existing datasets, which do not provide those critical data characteristics.
• The dataset is helpful for network administrators and network security researchers to analyze, evaluate and develop a new attack detection model based on the simultaneous characteristic that was occurring from several sensors’ detection at the same time.
• The dataset can be extended to a parallel detection model for more complex botnet activity detection. This parallel detection handles distributed bot attacks, which often occur in a real system. Three sub-datasets are obtained explicitly from different sensors containing normal and various botnet activities. Additionally, a sub-dataset is a combination of those three sub-datasets. Thus, it can be a knowledge database comprising botnet attack pattern behaviors.

## 1 Data Description

The dataset simulates botnet attacks using botnet activities described in CTU-13 [1] and NCC [2]. The simulation extracts all scenarios from those two datasets to determine attack activities, attack phases, and the time difference between attacks and normal activities [3], leading to four scenarios represented by the corresponding sub-datasets. Additionally, it is assumed that this simulation employs three sensors. This dataset is intended to be a basis for developing a distributed botnet detection model, requiring data sources with simultaneous botnet attack activity. It is an attack activity carried out by more than one type of botnets at the same time.

**Fig. 1:**
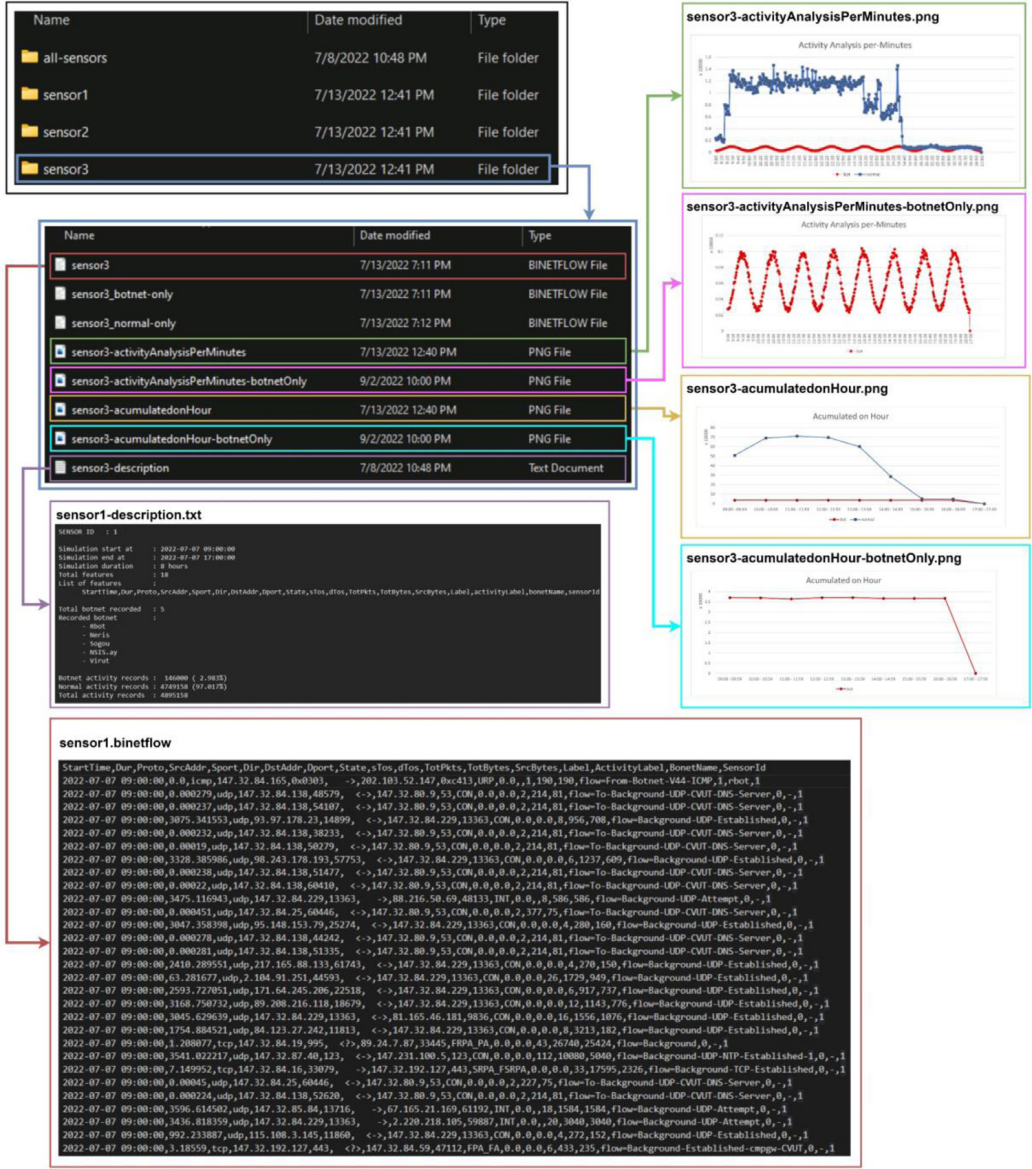
Examples of the dataset.

The first scenario consists of five botnet types: Rbot, Neris, Sogo, NSIS.ay, and Virut, which are detected by sensor 1. The second scenario has four botnet types: Rbot, Neris, Menti, and Virut, collected by sensor 2. The third scenario utilizes sensor 3, resulting in five botnet types: Rbot, Neris, Murlo, NSIS.ay, and Virut. Lastly, the fourth scenario combines outputs from those three sensors. An example of the output of sensor 1 is depicted in Fig. 1. In more detail, a description of scenarios is provided in Table 1. Each sensor's output is stored in a folder containing three bidirectional network flow (binetflow) files, a file in *.txt format describing the dataset, and four *.png image files showing the sensor's features. Let n be id of sub-dataset (sensor), the data can be described as follow.

• **sensor**n**-acumulatedonHour.png** illustrates the number of botnets and normal activities that accumulated every one hour in those specific sensors in our dataset.
• **sensor**n**-acumulatedonHour-botnetOnly.png** illustrates only the number of botnet activities accumulated every hour in our dataset's specific sensors.
• **sensor**n**-activityAnalysisPerMinutes.png** illustrates the number of the botnet and normal activities every minute in those specific sensors in our dataset.
• **sensor**n**-activityAnalysisPerMinutes-botnetOnly.png** illustrates the only number of botnet activities every minute in those specific sensors in our dataset.

**Table 1:** Simulation scenario for each sensor.

Sensor Id	Bot Name	Botnet Scenario Source (CTU-13 / NCC)	Type of Attack	Duration
1	Rbot	3,10	IRC, PS, DDoS, US	8 h
	Neris	9	IRC, SPAM, CF, PS	8 h
	Sogo	7	HTTP	8 h
	NSIS.ay	12	P2P	8 h
	Virut	5,13	SPAM, PS, HTTP	8 h
2	Rbot	10,11	IRC, PS, DDoS, US	8 h
	Neris	1,2,9	IRC, SPAM, CF, PS	8 h
	Menti	6	PS, HTTP	8 h
	Virut	13	SPAM, PS, HTTP	8 h
3	Rbot	3, 4	IRC, PS, DDoS, US	8 h
	Neris	1,2	IRC, SPAM, CF, PS	8 h
	Murlo	8	PS	8 h
	NSIS.ay	12	P2P	8 h
	Virut	5	SPAM, PS, HTTP	8 h

**Fig. 2(a):**
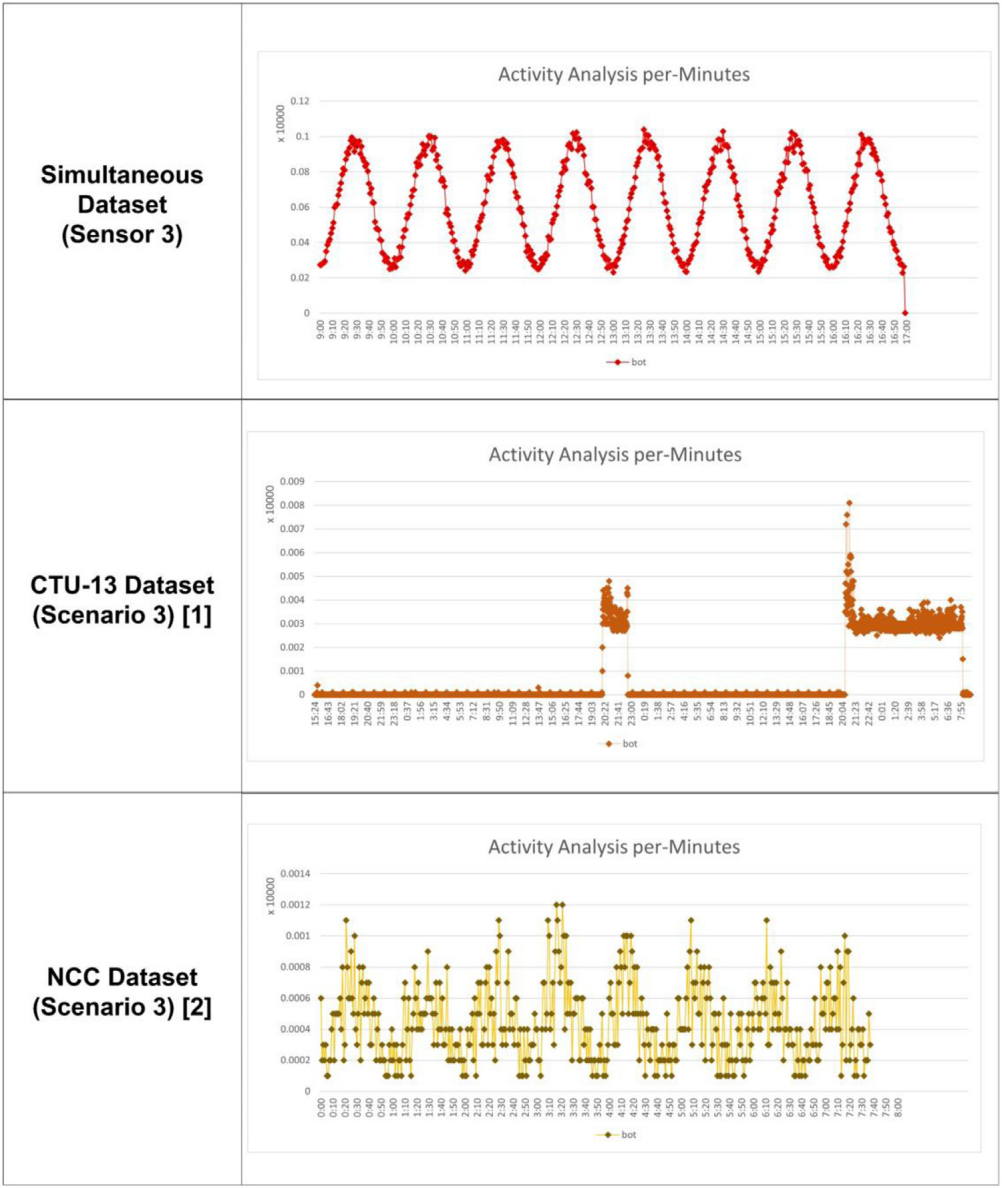
Comparison between the generated dataset, CTU-13 and NCC for scenario 3, per minutes.

**Fig. 2(b):**
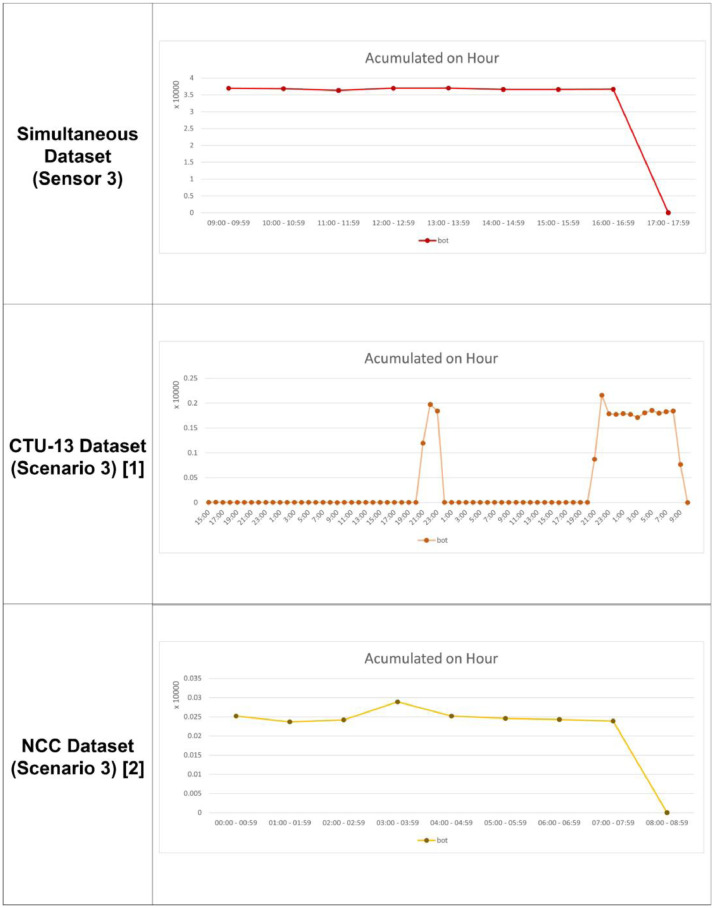
Comparison between generated dataset, CTU-13 and NCC for scenario 3, per hours.

The proposed dataset shown in Table 2 comprises 18 features as network headers representing network traffics. Among those features, three new features are extracted from the existing 15 features in CTU-13 and NCC datasets: ActivityLabel, BotnetName, and SensorId, describing the botnet name, activity label, and sensor id recording activities, respectively. The ActivityLabel has two possible values: 0 or 1, which indicate the network traffic is normal or botnet activities, respectively. The BonetName feature contains the attacking botnet's name; if it is a normal activity, the field is blank (-). SensorId is the identifier of the sensor recording network traffic.

**Table 2:** Details of each feature identifying network traffic.

Feature Name	Description	Value
StartTime	Activity time recorded/executed	Date Time Format, (YYYY-MM-DD HH:mm:ss)
Dur	Duration of the activity when executed and using resources	Integer (last time record - start time record)
Proto	Protocol that used in the transaction	String (TCP, UDP, etc.)
SrcAddr	Source IP address, source address of network activity	String (IP version 4)
Sport	Source Port, Port used in performing network activities	Integer (1507, 2609, etc.)
Dir	Direction, the direction of communication carried out	Categorical (->, <-, <->, etc.)
DstAddr	Destination IP address, address of network activity communication target in the form of IPv4	String (IP version 4)
Dport	Destination Port, Port used by the communication target	Integer (1507, 2609, etc.)
State	Information status of the data stream	Categorical (SRPA_SPA, FSPA_FSPA, etc.)
sTos	Source Type of Service	Float
dTos	Destination Type of Service	Float
TotPkts	All Transactions in *packet*	Integer
TotBytes	Overall Transaction in *bytes*	Integer
SrcBytes	Transactions in *bytes* from activity source to destination	Integer
Label	Network traffic labels	String (flow=Background, etc.)
ActivityLabel	Activity label, a binary value	Binary value, 1 for botnet and 0 for normal activity
BonetName	Name of the botnet performing the activity	String (Rbot, Neris, -, etc.)
SensorId	The identity of the sensor that records network activity	Integer (1, 2, 3)

This dataset has simultaneous botnet activity and more advanced characteristics in terms of attack intensity and the number of botnet types in one sub-dataset than the sporadic attacks on CTU-13 and periodic attacks on NCC datasets. The botnet activities tend to peak at random periods in sporadic characteristics. Besides, it peaks at a relatively constant time interval in the periodic characteristics [4]. Several botnets carry out attacks at the same time with simultaneous characteristics, which are substantially more intense than sporadic and periodic attacks. Most detection systems, especially models that use clustering and deep learning techniques, consume many resources, causing problems when the detection is carried out at the same time in a short time frame [5,6]. The proposed dataset has the characteristics of simultaneous attacks in a short period, so the security system must survive resource problems when dealing with botnet attacks. Different sensors parallelly detect the same type of bots or attack behavior with a simultaneous activity, which the CTU-13 and NCC datasets do not have. The CTU-13 dataset only records botnet activity without considering the number of sensors. Moreover, it focuses on one botnet type in one attack scenario. This has made CTU-13 cannot be used for the parallel botnet activity detection model. On the other side, NCC is designed to evaluate only associated actions, focusing on obtaining periodic activity on grouped botnets. In this research, these CTU-13 and NCC disadvantages are combined to produce simultaneous datasets. Table 3 shows a description of the proposed dataset with simultaneous attack characteristics, and Fig. 2(a), Fig. 2(b) compares the proposed dataset to the CTU-13 and NCC datasets with per minute analysis.

**Table 3:** Detail activity recorded for each sensor.

Sensors	Bot Activity	Bot Activity (%)	Normal Activity	Normal Activity (%)	Total Activity
1	146,000	2.983	4,749,158	97.017	4,895,158
2	364,000	6.069	5,634,133	93.931	5,998,133
3	294,000	7.566	3,591,792	92.434	3,885,792
All-Sensors	804,000	5.440	13,975,083	94.560	14,779,083

## 2 Experimental Design, Materials and Methods

**Fig. 3:**
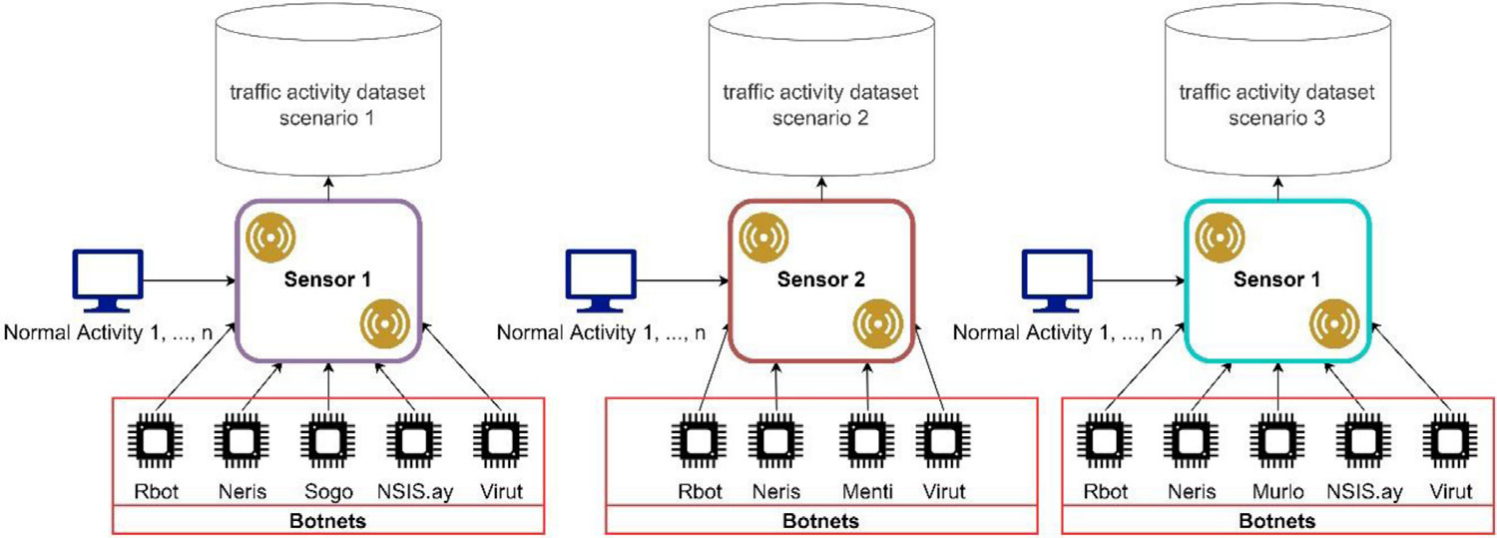
Simulation topology.

**Fig. 4:**
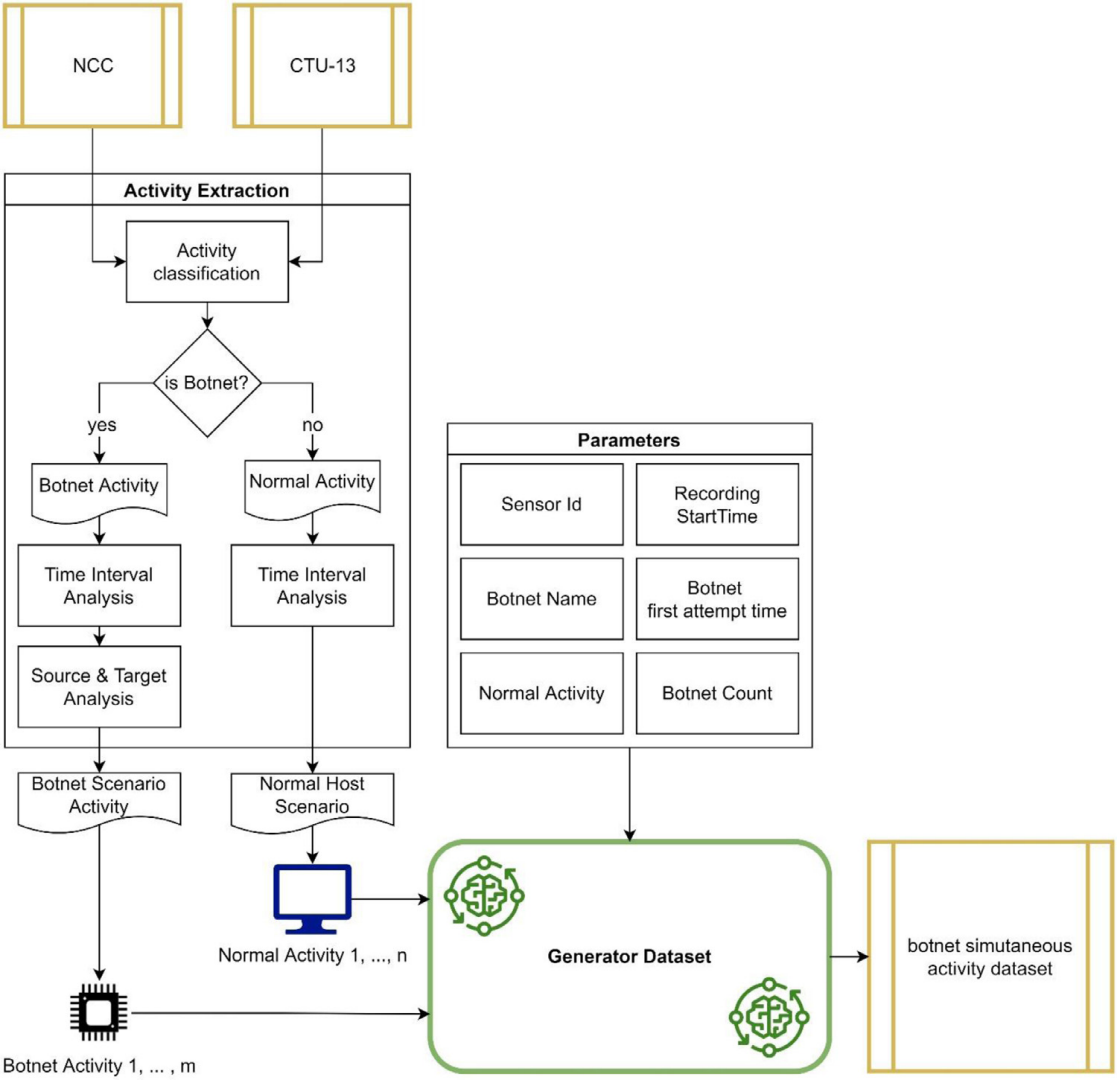
Dataset generation methods.

The topology of botnet attack activities is illustrated in Fig. 3. The simulation has three sensors, each consisting of four to five botnet types. Bot activity is obtained by extracting bot behavior from scenario 1 to 13 datasets on CTU-13 and NCC [1,2]. The extraction process is carried out to analyze bot attack behavior and normal behavior [3], whose extraction path is shown in Fig. 4.

All scenarios in the CTU-13 and NCC datasets are extracted to obtain attack activity, attack phase, and the time difference between attack and normal activity. Normal data are recorded sequentially, while the bot attack activities are further analyzed, starting by determining the time difference between attacks. Targeted attacks, such as DDoS, tend to flood network traffic for a specific period [7,8]. Therefore, the time difference between attacks must be monitored to identify a chain attack activity and a follow-up attack. Furthermore, attack such as SPAM is unique; this assault activity is more constant in the distance between attacks. After examining the time gap between attacks, the following analysis finds the attack source and target. This analysis is utilized to generate a new set of bot-to-target attacks. All findings are saved as a set of new attack stages based on the characteristics of each botnet [1]. Finally, the BotnetName feature is included to explain where the botnet attack activity originated.

The simulation process accepts several parameters, as described in Table 4. The attack simulation starts by adding a particular botnet to each sensor. Each attack activity is carried out in accordance with the previously extracted data and is organized depending on the attack stage, the interval between attacks, and the attack's characteristics. The simulation procedure is conducted simultaneously by adding normal activities sequentially.

**Table 4:** Parameters of dataset generator.

Name of Parameter	Description	Value
Sensor Id	Value that identifies each sensor	Sensor1, sensor2, sensor3
Botnet Name	Name of botnet that included for each sensor	String (Rbot, Virut, etc.)
Recording Start & End Time	The start time of network traffic recording in the dataset generation process using the simulation method	Date Time Format, (2022/07/07 09:00:00.000 - 2022/07/07 17:00:00.000)
Botnet First Attempt Time	The beginning of a botnet sequential attack activities chain.	Date Time Format, (2022/07/07 09:00:00.000)
Number Of Bots	Number of source bots performing attacks.	Sensor 1: 27 bots Sensor 2: 27 bots Sensor 3: 9 bots
Normal Activities Sources	Source of normal activity to be recorded on the sensor. Normal activity was obtained from several scenarios in the CTU-13 and NCC datasets.	Number of Normal Host:- Sensor 1: 723,628 hosts- Sensor 2: 861,587 hosts- Sensor 3: 448,298 hosts

**Fig. 5:**
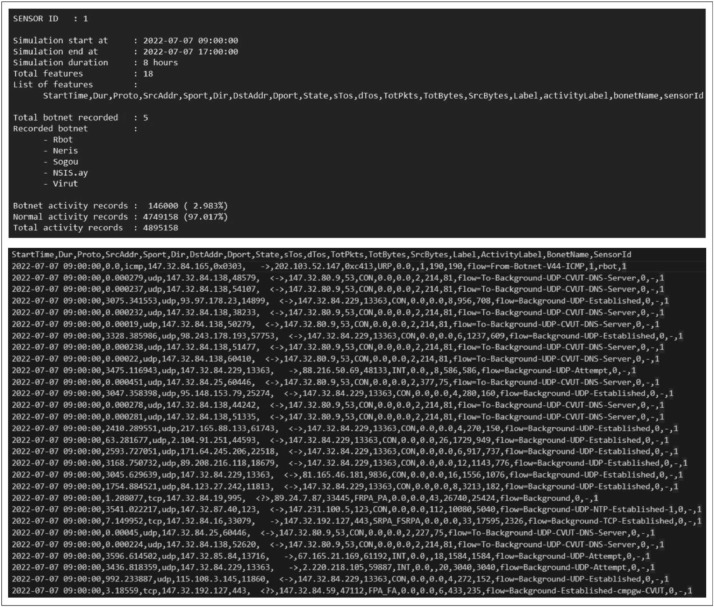
Result of generating dataset process.

The simulation results are saved as a bidirectional network flow (.binetflow) file representing data obtained from a specific sensor. The process is completed by combining those three binetflow files from different sensors, and all resulting datasets are shown in Fig. 5.

## Ethics Statements

This work does not involve things like human subjects, animal experiment and data collection from social media platforms.

## CRediT authorship contribution statement

**Muhammad Aidiel Rachman Putra:** Conceptualization, Software, Formal analysis, Visualization, Writing – original draft. **Dandy Pramana Hostiadi:** Conceptualization, Validation, Methodology, Investigation, Formal analysis, Writing – review & editing. **Tohari Ahmad:** Supervision, Conceptualization, Writing – review & editing, Funding acquisition.

## Declaration of Competing Interest

The authors declare that they have no known competing financial interests or personal relationships that could have appeared to influence the work reported in this paper.

## Data Availability

NCC-2 Dataset: Simultaneous Botnet Dataset (Original data) (Mendeley Data).
